# Panax Ginseng C.A.Mey. as Medicine: The Potential Use of Panax Ginseng C.A.Mey. as a Remedy for Kidney Protection from a Pharmacological Perspective

**DOI:** 10.3389/fphar.2021.734151

**Published:** 2021-08-26

**Authors:** De Jin, Yuqin Zhang, Yuehong Zhang, Liyun Duan, Rongrong Zhou, Yingyin Duan, Yuting Sun, Fengmei Lian, Xiaolin Tong

**Affiliations:** Department of Endocrinology, Guang’anmen Hospital, China Academy of Chinese Medical Sciences, Beijing, China

**Keywords:** ginseng, diet supplements, diabetic kidney disease, pathological phenotypes, pharmacological perspective -3 -

## Abstract

Panax ginseng C.A.Mey. has been widely consumed as food/diet supplements from natural sources, and its therapeutic properties have also aroused widespread concern. Therapeutic properties of Panax ginseng C.A.Mey. such as anti-inflammatory, ameliorating chronic inflammation, enhancing the immunity, resisting the oxidation again, and regulating the glucose and lipid metabolism have been widely reported. Recent years, lots of interesting studies have reported the potential use of Panax ginseng C.A.Mey. in the management of DKD. DKD has become the leading cause of end-stage renal disease worldwide, which increases the risk of premature death and poses a serious financial burden. Although DKD is somehow controllable with different drugs such as Angiotensin-Converting Enzyme Inhibitors (ACEI), Angiotensin Receptor Blockers (ARB) and lowering-glucose agents, modern dietary changes associated with DKD have facilitated research to assess the preventive and therapeutic merits of diet supplements from natural sources as medicine including Panax ginseng C.A.Mey. Findings from many scientific evidences have suggested that Panax ginseng C.A.Mey. can relieve the pathological status in cellular and animal models of DKD. Moreover, a few studies showed that alleviation of clinical phenotype such as reducing albuminuria, serum creatinine and renal anemia in DKD patients after application or consumption of Panax ginseng C.A.Mey.. Therefore, this review aims to discuss the effectiveness of Panax ginseng C.A.Mey. as medicine for targeting pathological phenotypes in DKD from a pharmacological perspective. This review will provide new insights into the potential understanding use of Panax ginseng C.A.Mey. in the management of DKD in clinical settings.

## Introduction

An ancient proverb states “Food/Diet Supplements from Natural Sources as Medicine”, and this adage is supported by the results of the Global Burden of Disease (GBD) Study 2017, which showed that dietary risk factors and poor diets contributed to 11 million premature deaths and 255 million disability-adjusted life-years. Another study reported that poor diets (such as ultra-processed foods, soft drinks, poultry or fish nuggets and salty snacks are associated with an increased risk of diabetes and its complications (Srour et al., 2020). These staggering data show that suboptimal diets may lead to more deaths and highlight the “Food/Diet Supplements from Natural Sources as Medicine” as a strategy for improving poor diet, combating the burden of non-communicable diseases, including diverse kidney diseases (KD). “Food/Diet Supplements from Natural Sources as Medicine” could potentially have positive effects on kidney protection (KP) (Stenvinkel et al., 2020).

**FIGURE 1:**
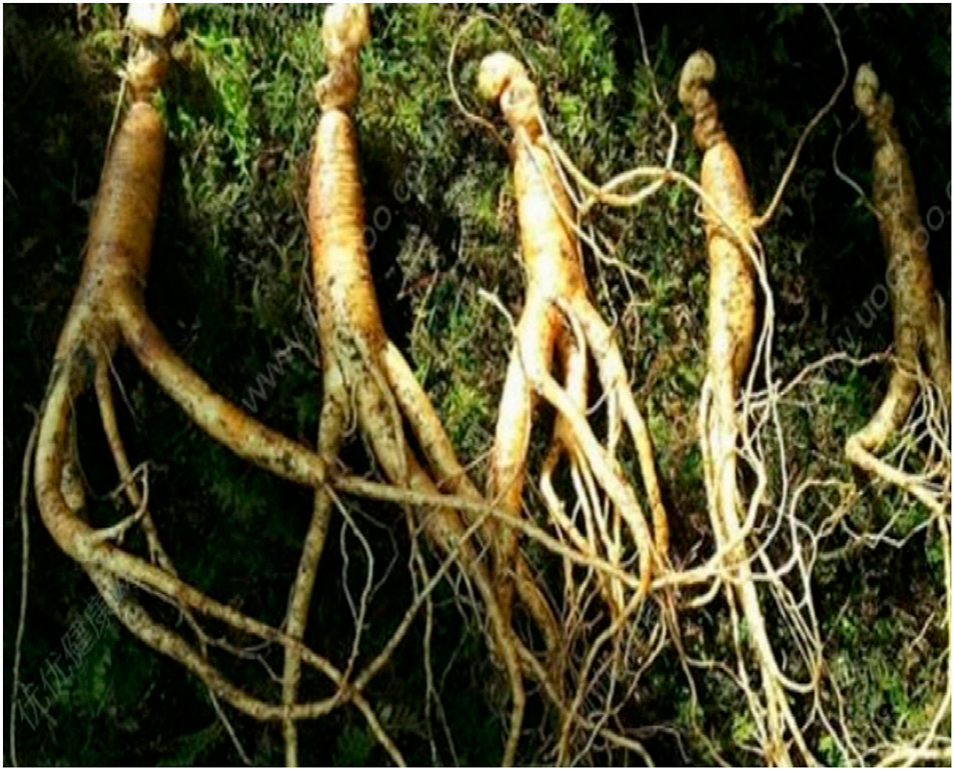
The photo of Ginseng. The photo of Ginseng collected from Jilin province, one of major production regions of Ginseng in China.

Panax ginseng C.A.Mey. has been widely consumed as food/diet supplements from natural sources, and its therapeutic properties have also aroused widespread concern (Figure 1) (Fan et al., 2020a). Therapeutic properties of Panax ginseng C.A.Mey. such as anti-inflammatory, altering the composition and metabolism of the microbiota, ameliorating chronic inflammation, enhancing the immunity, resisting the oxidation again, and regulating the glucose and lipid metabolism have been widely reported (Cao et al., 2019; Jang et al., 2019; Liu et al., 2019, 2020; Xue et al., 2019; Huang et al., 2020; Quan et al., 2020; Xu et al., 2020). Intriguing new data suggest that Panax ginseng C.A.Mey. could minimize renal injury by inhibiting oxidative stress, inflammatory responses, epithelial-mesenchymal transition, and fibrosis (Liu et al., 2020; Shi et al., 2020; Xie et al., 2020; Zhu et al., 2020; Li et al., 2021). This finding is pertinent to the pathologic phenotype of KD, which is characterized by features of destruction of the glomerular filtration barrier involving podocyte, basement membrane and endotheliocyte (Thomas et al., 2015). Further, these pathological changes could contribute to the deterioration of renal function such as proteinuria, increased serum creatinine, changes in glomerular filtration rate and renal anemia (Thomas et al., 2015). Clinically, Panax ginseng C.A.Mey. also has been shown to possess beneficial effects in the treatment of KP (Lang et al., 1998; Zhao et al., 2007).

Over the last decade, promising advancements have been made in the mechanisms and clinical outcomes of Panax ginseng C.A.Mey. on KD. Here, we are focusing on a discussion of Panax ginseng C.A.Mey. associating with KP. In addition, the possible active ingredients within Panax ginseng C.A.Mey. responsible for KP are elucidated. Table 1 summaries the kidney protection performances of ginsenosides in preclinical studies. These contents will be overviewed in detail in the following sections.

**TABLE 1:** Ginsenosides tested in animal or cellular studies for human kidney-related diseases.

Model	Ginsenosides	Animal/cell type	Therapeutic mechanism	References
Targeting podocytes	Panax notoginseng saponins	STZ rats and podocyte cells	Inhibiting the podocyte cell apoptosis *via* reducing oxidative stress	Sun et al. (2011)
	Panax notoginseng saponins	STZ rats	Increasing the mRNA expression of nephrin, α3β1 integrin proteins	Zhou et al. (2014)
Targeting glomerular basement membrane nephritis	Rg1	STZ rats, podocyte cells and basement membrane cells	Inhibiting glomerular basement membrane nephritis through regulating the nuclear factor E2-related factor 2 (Nrf2) pathway	Guo et al. (2019b)
	Rg1	STZ rats, podocyte cells and basement membrane cells	Suppressing inflammation, oxidative stress *via* activating Nrf2 signal pathway	Li et al. (2020c), Alaofi (2020), Li et al. (2020b)
Targeting glomerular endothelial barrier	Rg1	HUVEC	Reducing the heparanase mRNA and transendothelial resistance and transendothelial albumin pass rate	Zhu et al. (2019)
Targeting glomerular mesangial cells	Rg1	HGMC	Inhibition of the TGF-β/Smad signaling pathway and glycosylation end products	Zhao (2018)
	Rb1	HBZY-1	Promoting the proliferation of mesangial cells, inhibiting cell apoptosis and Caspase-3 expression	Tang et al. (2013)
	Rg1	HGMC	Reducing the number of apoptotic cells, the activity of extracellular lactate dehydrogenase and block the cell cycle of human glomerular mesangial cells in G1 phase, and downregulate the mRNA and protein expression level of Cyclin-dependent kinase 4 (CDK4)	Wu and Wang (2015)
Targeting renal tubular cells	Rb1	HK-2	Activating the PI3K/AKT pathway and inhibiting the NF-KB pathway	Ni et al. (2017)
	Rb1	HK-2	Upregulating the expression of proliferating cell nuclear antigen	Yang et al. (2015)
	Rb1	Rats model reduced by cunilateral ureteral obstruction	Inhibiting the transcription and activation of transforming growth factor-β1	Xie et al. (2008), Li et al. (2015a)
Targeting chronic kidney disease	Rd	Rats model reduced by ischemia-reperfusion	Preventing oxygen free radicals from attacking the cell membranes	Yokozawa et al. (1998)
	Rb1	CKD patients	Reducing the levels of inflammatory factors TNF-a and 1L-6, and creatinine level	Xu et al. (2017)
Targeting diabetic kidney disease	Rb1	HGMC	Inhibiting the phosphorylation levels of P38 MAPK, JNK/SAPK and Akt	Park et al. (2010)
	Rb1	Rats model reduced by streptozotocin	reduce serum creatinine and urea nitrogen, mesangial hyperplasia of the glomerulus, and dilatation of renal tubules	Zhang et al. (2008), Zhao et al. (2008), Koizumi et al. (2013)
Targeting acute kidney injury	Rb1	Glycerol-induced acute r enal failure	Activating heme oxygenase (HO-1), Nrf2, and reducing ROS peroxidation	Sun et al. (2013), Sun, (2014)
	Rb1	Unilateral ureteral infarction in rats	Inhibit interstitial fibrous tissue, including tubular tissue damage and collagen deposition *via* reducing TGF-β1, HO-1and 8-OHdG	Xie et al. (2009a)
	Rb1	Renal artery ischemia of white rabbits	Downregulating the expression of Bcl-2 and Bax to inhibit apoptosis	Zhu et al. (2009)
	Rb1	Unilateral ureteral infarction in rats	Reducing the content of MDA and increase the activity of SOD in renal tissue	Sun et al. (2018)
Targeting renal senescence	Rb1	SAMP8 mice	Inhibiting the expression of the fibrinogen TGF-β1 and upregulating the renal protective factor BMP-7	Docherty et al. (2019)
Targeting renal fibrosis	Rg1	UUO animal models	Decreasing a-SMA and E-cadherin expression in the obstructed kidney models and reduce TGF-β1 induced by rat tubular cells	Xie et al. (2008), Xie et al. 2009b, Li et al. (2015a)
	Rg1	UUO animal models	Reverse EMT and renal interstitial fibrosis via targeting the TGF-β1/Smad pathway	Li et al. (2018)
	Ginseng extract	Cyclosporine A animal models; HK-2 cells	Improve renal function and inhibit apoptotic cell death	Doh et al. (2013), Liu et al. (2015)
	Ginseng extract	Cyclosporine A animal models	Targeting the Akt/mTOR pathway	Lim et al. (2014)
	Ginsenosides	Diabetic nephropathy rats	Protect kidney function *via* enhancing SIRT1 and suppressing inflammation	Du et al. (2016)

## Potential Bio-Active Compounds of Panax Ginseng C.A.Mey. in Kidney Protection

**FIGURE 2:**
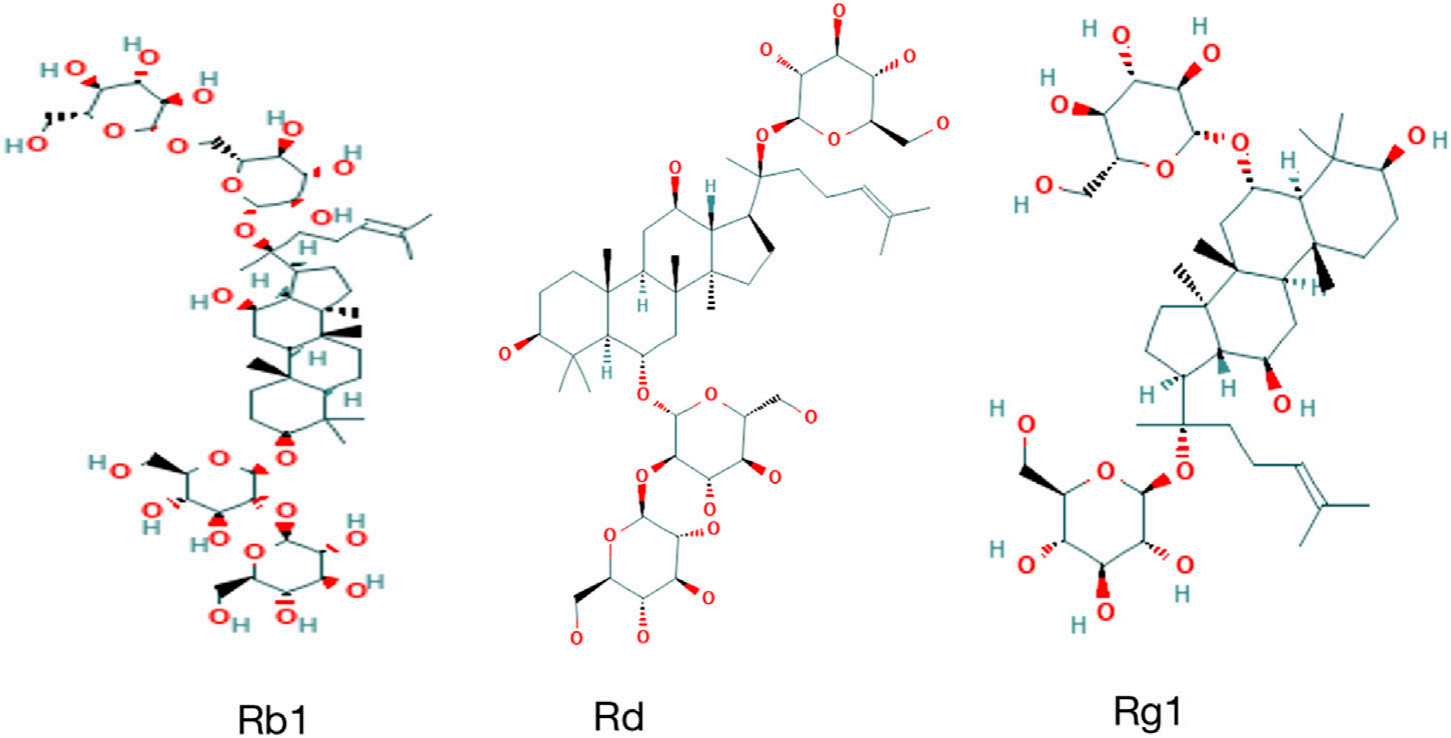
Chemical structure of ginsenosides in this article.

At present, more than 150 monomers have been identified and isolated from the roots, stems, leaves, flowers, and fruits of Panax ginseng C.A.Mey., of which more than 30 ginsenoside monomers have been identified in Panax ginseng C.A.Mey. as effective ingredients (Chang-Xiao and Pei-Gen, 1992; Nah, 1997; Wong et al., 2015). Panax ginseng C.A.Mey. triol (PT) saponins are also the main representative components of ginsenosides Rg1, Rd, Rb1, which have a high content of active parts and strong activity (Wong et al., 2015). Here, the compounds having potential beneficial effects on kidney protective function are highlighted (Figure 2).

## Protective Effect of Panax Ginseng C.A.Mey. on Renal Innate Cells

The preponderance of evidence supports the obvious: Panax ginseng C.A.Mey. can relieve renal innate cells damage and has a protective effect of Panax ginseng C.A.Mey. on the glomerular filtration barrier. The kidney filtration barrier is surrounded by three layers: 1) a fenestrated endothelium, 2) a basement membrane, and 3) the podocytes. The glomerular filtration barrier can effectively prevent albumin and larger molecular weight substances in the plasma from entering the urine. The changes in the structure and function of the glomerular filtration barrier caused by various reasons are the pathophysiological basis of proteinuria (Blaine and Dylewski, 2020). Studies have shown that proteinuria reflects not only kidney damage but also an independent risk factor leading to the progression of kidney disease (Webster et al., 2017). Therefore, understanding the molecular structure and function of the glomerular filtration barrier are essential for delaying the progression of KD. Here, we highlight Panax ginseng C.A.Mey. on kidney protection in the kidney filtration barrier and innate renal cells.

### Improving Podocyte Injury

Podocytes are epithelial cells that regulate the glomerular filtration barrier, which characterized by actin-rich foot processes that reside on the glomerular basement membrane (GBM) (Leeuwis et al., 2010). The disappearance of podocyte foot processes is a sign of podocyte damage and proteinuric kidney disease, accompanied by changes in podocyte protein expression and reorganization of the actin cytoskeleton (Fan et al., 2021). Panax notoginseng is the main active ingredient of Panax notoginseng (Burkill) F.H.Chen including Ginsenoside, (Rg1, Rg2, Rb1, Rb2, Rb3, Rc, Rd, Re, Rh, F2), Panax notoginosides (R1, R2, R3, R6, Fa, Fc, Fe, R4). In recent years, the role of PNS in improving podocyte damage has received widespread attention. Sun et al. (2011) reported that in the STZ-induced Diabetic Nephropathy (DN) rat model and the podocyte cell model stimulated by high glucose, the podocyte apoptosis might be related to oxidative stress, and PNS can improve oxidative stress-related indicators *in vivo* and *in vitro*, as well as downregulate the expression of the apoptosis marker protein (caspase-3). In addition, Zhou et al. observed the protective effect of PNS on the podocytes of DN rat. The findings showed that after 10 weeks of PNS intervention, the number of podocytes, mRNA expression of nephrin, α3β1 integrin proteins in the kidney tissue have been significantly improved accordingly (Zhou et al., 2014). These studies demonstrate promising evidence that PNS can improve podocyte damage and delay the progression of DN.

### Anti-inflammatory Effects on Glomerular Basement Membrane

The glomerular basement membrane (GBM) plays a key role in the maintenance of the structural integrity of the glomerular capillaries (Kalluri, 2003). Changes in the structural composition and thickness of the glomerular basement membrane can affect the occurrence and development of diverse kidney diseases. Ginsenoside Rg1 inhibits glomerular basement membrane nephritis through regulating the nuclear factor E2-related factor 2 (Nrf2) pathway and reduces the inflammation and apoptosis of podocytes induced by IL-1β, which inhibits the expression of Nrf2. Rg1 can increase the expression of Nrf2 (Guo et al., 2019b). Nrf2 is an important transcription factor that regulates the oxidative stress response of cells, and it is also a central regulator that maintains the intracellular redox homeostasis, reduces cell damage caused by reactive oxygen species and electrophiles, and maintains the body’s redox homeostasis (Li et al., 2020c). Nrf2 activation can suppress inflammation, oxidative stress and kidney tissue impairment (Alaofi, 2020; Li et al., 2020b). Thence, Rg1 could be regarded as an activating agent of Nrf2 for prevention strategy for DN progression.

### Protection of Glomerular Endothelial Barrier Function

Over the past decade, the preponderance of evidence supports that Panax ginseng C.A.Mey. can improve vascular endothelial function in several diseases (Nabavi et al., 2015). The destruction of the endothelial barrier is critical for vascular complications related to diabetes, and damage to the endothelial glycocalyx has been shown to be involved in this process (Fu et al., 2015). Rg 1 is the main active ingredient extracted from Panax notoginseng and has been widely used to prevent vascular damage. Recent research (Zhu et al., 2019) showed that high glucoscouldan induce endothelial glycocalyx disorders and increase the expression of heparanase mRNA in HUVEC, and Rg1 treatment can reverse this expression. In addition, after high glucose stimulation, Rg1 treatment can reduce transendothelial resistance and transendothelial albumin pass rate. It is worth noting that Rg1 has a protective effect on endothelial barrier dysfunction.

### Inhibition Apoptosis of Glomerular Mesangial Cells

Glomerular mesangial cells (MCs) are major components of the glomerular mesangium, hold the capillary arteries and connect them with the juxtaglomerular apparatus. More and more evidences are suggesting the abnormal growth of MCs is an early event in various glomerular diseases (Yoon et al., 2020; Wan et al., 2021). Targeting glomerular mesangial cells as the research object and screening for suitable drugs to repair the damage caused by oxidative stress and inflammation have important scientific research significance and clinical value. Rg 1 has been demonstrated to have a wide range of pharmacological properties, such as anti-inflammatory, anti-oxidation, anti-ageing, anti-fatigue and anti-tumour activities (Shen et al., 2017; Fan et al., 2020b). Rg1 has an anti-inflammatory effect, and the anti-inflammatory effect may be related to the inhibition of the TGF-β/Smad signaling pathway (Zhao 2018). A certain concentration of ginsenoside Rg 1 can inhibit glycosylation end products (AGEs) caused by TNF-α And cyclooxygenase-2 (COX-2) (Zhao. 2018). Notably, Rg 1 can promote the proliferation of mesangial cells and inhibit cell apoptosis, which may be related to the promotion of Bcl-2 and the inhibition of Caspase-3 expression (Tang et al., 2013). Besides, in the oxidative stress injury model of human glomerular mesangial cells induced by H_2_O_2_, Rg 3 can reduce the number of apoptotic cells damaged by H_2_O_2_, the activity of extracellular lactate dehydrogenase (LDH), and the content of malondialdehyde (MDA) as well as block the cell cycle of human glomerular mesangial cells in G1 phase, and downregulate the mRNA and protein expression level of Cyclin-dependent kinase 4 (CDK4) (Wu and Wang, 2015). These evidences reflect that Rg 1 exerts renal protection by inhibiting the apoptosis of glomerular mesangial cells.

### Inhibition Epithelial-Mesenchymal Transdifferentiation of Renal Tubular Cells

Epithelial-mesenchymal transition (EMT) is a biological process that directs changes in cell states along the epithelial versus mesenchymal axes, which is a hallmark of tubulointerstitial renal fibrosis (Chen et al., 2020). In addition, the nuclear factor-KB (NF-KB) pathway mainly mediates the inflammatory response, which is associated with activation for phosphatidylinositol 3-kinase (PI3K) and serine/threonine kinase (Akt) pathway (Yang et al., 2019). These signal pathways play a key role in the EMT.

Evidence from several *in vitro* studies has suggested that Rg 1 plays a positive role in improving EMT of renal tubular cells. NI et al. found that ginsenoside Rg l protects the human renal tubular epithelial cell line HK-2 from lipopolysaccharide (LPS)-induced inflammation and apoptosis *via* activating the PI3K/AKT pathway and inhibiting the NF-KB pathway, thereby inhibiting the EMT in renal tubular cells (Ni et al., 2017). This result was confirmed by Yang et al. They used urine protein to induce renal tubular epithelial cell damage and reduce the cell survival rate. After administration of Rg l, the cell survival rate was improved. And the expression of proliferating cell nuclear antigen (PCNA) protein and PCNA mRNA were upregulated (Yang et al., 2015). The results from the *in vitro* studies were confirmed using an *in vivo* model. In a rat model of renal interstitial fibrosis induced by unilateral ureteral obstruction (UUO), Rg1 inhibits the transcription and activation of transforming growth factor-β1 (TGF-β1) and inhibits the transdifferentiation of tubular epithelial myofibroblast (Tubular epithelial myofibroblast transdifferentiation, TEMT) (Xie et al., 2008; Li et al., 2015a). These data provided evidence that the tubular epithelial cells were responding to Rg1treating EMT.

## Role of Panax Ginseng C.A.Mey. in Different Nephropathy

Kidney diseases worldwide prevalence have reached epidemic proportions globally in the last few decades. Several drugs that provide kidney function protection have been widely used. Unfortunately, drug resistance and adverse events have limited their applicability in the clinic. Recently, Panax ginseng C.A.Mey. and Panax ginseng C.A.Mey. extracts have attracted much attention as effective and safe alternative drugs for kidney diseases. Here, we will comprehensively summarize the current studies of Panax ginseng C.A.Mey. in different kidney diseases.

### Chronic Kidney Disease

Chronic Kidney Disease (CKD) usually has concealed onset and gets progressively worse, followed by an increasing burden of hypertension, atherosclerosis, calcium and phosphorus metabolism disorders, renal anaemia and other complications, which is a cause of substantial healthcare expenditure (Chen et al., 2019; Tonelli and Dickinson, 2020). Oxidative stress is known as the main mechanism of CKD, and antioxidants have potential value in the treatment of CKD (Podkowińska and Formanowicz, 2020). Most studies have proved that ginsenosides have good anti-oxidation and free radical scavenging functions *in vivo* and *in vitro* (Jung et al., 1998; Kim et al., 2000; Kim et al., 2013). Some scholars investigated the effect of Rd in rats with ischemia-reperfusion. Findings indicate that ginsenoside-Rd could affect cultured proximal tubule cells subjected to hypoxia-reoxygenation, probably by preventing oxygen free radicals from attacking the cell membranes (Yokozawa et al., 1998). This was demonstrated in a preliminary clinical trial by Xu et al. CKD patients on oral Rb1 (500 mg) were found to be more likely to have a lower level of creatinine than among those on the placebo group. Additionally, compared with the placebo group, the of the active Oxygen is significantly improved, and the levels of inflammatory factors TNF-a and 1L-6 are significantly reduced in the Rb1 group (Xu et al., 2017).

### Diabetic Kidney Disease

Diabetic kidney disease (DKD) is one of the common and serious long-term complications of hyperglycemia, and long-term glucose metabolic disorder is the main cause of DKD (Barrera-Chimal and Jaisser, 2020). The clinical manifestations are reduced glomerular filtration rate, followed by microalbuminuria, elevated arterial blood pressure, and fluid retention, leading to renal failure (Matoba et al., 2019). In the primary mesangial cell model induced by high glucose, it was found that ginsenoside Rb1 mainly inhibited the phosphorylation levels of _P_38 MAPK, JNK/SAPK and Akt, and thus suppressed the expression of mesangial fibroconnectin induced by high glucose (Park et al., 2010). At animal levels, Rb1 can improve the quality of life of diabetic nephropathy induced by streptozotocin in rats, reduce serum creatinine and urea nitrogen, mesangial hyperplasia of the glomerulus, and dilatation of renal tubules, mainly *via* down-regulating mRNA and protein expression of MCP-1 mRNA and TGF-β1 mRNA in kidney tissue (Zhao et al., 2008; Zhang et al., 2008; Koizumi et al., 2013). In addition, clinically, strict glycaemia and blood pressure control can also slow the progression of DKD (Doshi and Friedman, 2017). Panax ginseng C.A.Mey. plays an important role in the improvement of hypertension, hyperglycaemia and lipid metabolism disorders. Rb1 can reduce the complications of diabetic nephropathy by reducing free fatty acids, promoting lipid metabolism, improving insulin resistance in obese mice, inhibiting the levels of TNF-a and IL-6 inflammatory factors, and the decomposition of adipocytes (Wang, 2011). Ginsenosides (Rg1, Rg3, Rb1 and compound K) act on the targets of Caspase-3, Bel-2, MDA, and SOD, reducing the gluconeogenesis, lipid metabolism, inflammatory and oxidation in diabetic nephropathy, which can be used as a potential adjunctive drug for the treatment of diabetes (Bai et al., 2018; Shao et al., 2020).

### Acute Kidney Injury

Acute kidney injury (AKI) is a common clinical critical illness, mainly manifested as the accumulation of metabolic substances and declined renal functions (Guo et al., 2019a). Renal ischemia and reperfusion injury (IRI) is a major cause of acute kidney injury (AKI) (Singbartl and Kellum, 2012). Ginsenosides are reported to have antioxidant and anti-inflammatory effects, as well as remit kidney damage caused by intestinal ischemia in mice. Rb1 can activate heme oxygenase (HO-1), Nrf2, and reduce ROS peroxidation damages and protect mitochondrial function to reduce kidney damages (Sun et al., 2013; Sun, 2014). In acute kidney injury mediated by unilateral ureteral infarction in rats, Rb1 can significantly inhibit interstitial fibrous tissue, including tubular tissue damage and collagen deposition *via* reducing TGF-β1, HO-1and 8-OHdG (Xie et al., 2009a). In the renal artery ischemia of white rabbits, Rb1 can downregulate the expression of Bcl-2 and Bax to inhibit apoptosis and reduce kidney damage (Zhu et al., 2009). Similarly, Sun et al. found that Rb1 can reduce the content of MDA and increase the activity of SOD in renal tissue, and then downregulate the expression of Caspase-3 in renal cells, thus alleviating the apoptosis of renal cells induced by ischemia and reperfusion to protect the kidney function (Sun et al., 2018).

### Renal Senescence

Progressive renal recession is a common phenomenon in the ageing process, and ageing-related declines in renal function are associated with a progressive loss of functioning nephrons (Fang et al., 2020). Glomerular basement membrane thickening, mesangial matrix hyperplasia, segmental glomerulosclerosis, renal tubular atrophy and tubulointerstitial fibrosis, arterial intimal fibrous thickening are the main histological features of renal ageing (Docherty et al., 2019). Ginsenoside Rbl can inhibit the expression of the fibrinogen TGF-β1 and upregulate the renal protective factor BMP-7 to reduce the abnormal accumulation of ECM components in the process of renal ageing, thereby harnessing tubular interstitial damage and glomerular sclerosis in the kidney ageing in SAMP8 mice (Docherty et al., 2019).

### Renal Fibrosis

Chronic kidney disease, as a global public health burden, has attracted great attention. So far, it has faced huge challenges due to the lack of effective treatment strategies (Yang et al., 2020). Renal fibrosis is an important pathological process from chronic kidney disease to end-stage renal diseases (Humphreys, 2018). Ginsenosides have shown to exert a renoprotective effect. Findings from Xie et al. showed that ginsenoside Rg1 could retard interstitial fibrosis in the UUO animal models (Xie et al., 2008). Intriguingly, Rg1 also was reported that Rg1 could decrease a-SMA and E-cadherin expression in the obstructed kidney models and reduce TGF-β1 induced by rat tubular cells, hinting that the potential mechanism might be partly related to the kickbacking of EMT (Xie et al., 2008; Xie et al., 2009b; Li et al., 2015a). Besides this, their results showed that Rg1 could reverse EMT and UUO-induced renal interstitial fibrosis *via* targeting the TGF-β1/Smad pathway (Li et al., 2018).

As a commonly used clinical immunosuppressant, cyclosporine A (CsA) has been widely prescribed for inhibiting rejection after organ transplantation. Many experimental studies showed that long-term use of CsA could contribute to progressive renal interstitial fibrosis, renal cell apoptosis, and immune cell infiltration (Wu et al., 2018; Lin et al., 2019). Findings from Doh’s group showed that Panax ginseng C.A.Mey. extract could effectively improve renal function and inhibit apoptotic cell death *in vivo* and vitro (Doh et al., 2013; Liu et al., 2015). Meanwhile, the Akt/mTOR pathway participating in Panax ginseng C.A.Mey. extract for the CsA animal model has been identified, which might be involved in autophagosome formation and autophagic aggregates (Lim et al., 2014). In diabetic nephropathy rats, the protective role of ginsenosides on diabetic nephropathy was also verified *in vivo* experiments. Du et al. suggested that Panax ginseng C.A.Mey. administration could protect kidney function *via* enhancing SIRT1 and suppressing inflammation in diabetic nephropathy rats (Du et al., 2016). In light of the above, ginsenosides were suggested as an important option during the treatment of renal fibrosis.

## Panax Ginseng C.A.Mey. for Kidney-Related Diseases in Clinical Trials

In recent years, accumulating evidence has revealed that Panax ginseng C.A.Mey. has beneficial effects against diabetes, obesity, stroke, and cardiovascular diseases (Kim, 2012; Rastogi et al., 2014; Zhang et al., 2014; Gui et al., 2016; Hong et al., 2020). However, fewer data are available in the field of Panax ginseng C.A.Mey. for kidney-related diseases in clinical trials. Here, we summarized this topic while adding some important updates and focused on the clinical trials of kidney-related diseases. Li et al. implemented a randomized single-blinded trial with an administration of American Panax ginseng C.A.Mey. compound liquor or American Panax ginseng C.A.Mey. liquor. Their results showed that Panax ginseng C.A.Mey was beneficial to improve microcirculation, reduce whole blood viscosity and decrease urinary albumin so as to retard the progress of DKD (Lang et al., 1998). The results agreed with the findings of CKD patients from Peng and Guo (2010) and Xu et al. (2017). Peng et al. focused on the Panax notoPanax ginseng C.A.Mey. for chronic renal failure (CRF), which the study showed that Panax notoPanax ginseng C.A.Mey. possessed such therapeutic effects as improving the renal function and lowering urine protein (Peng and Guo, 2010). The aim of Xu et al. study (Xu et al., 2017) was to evaluate the effects of Rb1 (500 mg daily oral administration) prospectively in patients with early CKD (stage 2 or 3) for 6 months. Findings showed that GS-Rb1 could present an antioxidant-based approach to slow the progression of CKD at the early stages (Xu et al., 2017). However, some studies concluded the opposite (Stavro et al., 2006; Kulaputana et al., 2007). Panax ginseng C.A.Mey. did not affect serum cystatin C level, 24-h BP and renal function (Stavro et al., 2006), as well as an ergogenic property on aerobic fitness enhancement in well-fit individuals (Kulaputana et al., 2007). The conflicting evidence outlined above might be due to the poor research quality employed. The reason can attribute to the following reasons: 1) the sample size of these studies was relatively small and results in weak statistical power. 2) patients with CKD form heterogeneous study populations, so that it may be more difficult to control for confounding. Especially, DKD population is highly heterogeneous in terms of comorbid illnesses and functional impairments. 3) Under population heterogeneity, selection of the features that affect the drug sensitivity has not been addressed in trials. These factors may have contributed to the inconsistency of these findings.

## Limitations and Future Perspectives

Ginsenosides as natural medicine have been widely approved to exert therapeutic effects in kidney-related *in vivo* and vitro. However, human clinical studies present several limitations. There are several existing obstacles concerning the utilization of ginsenosides or Panax ginseng C.A.Mey. before translation into clinical application is made possible. One of the most difficult handicaps is a lack of high-quality, evidence-based medical evidence for Panax ginseng C.A.Mey. treating kidney-related diseases. In respect of study population selection, the current research is still insufficient due to the focusing on a single participant (such as DKD, CKD); thus, further exploration (such as IgA nephropathy, Kidney ageing) is required. In terms of the selection of the indicators, the current study focuses on more serum creatinine levels, proteinuria, urea nitrogen, and physiological and biochemical parameters, less than estimated glomerular filtration rate (eGFR), urine albumin-creatinine ratio (UACR) and the composite of renal outcomes (annual rate of change in GFR, doubling of serum creatinine level or 50% reduction in GFR, end-stage renal disease).

In addition, CKD may have electrolyte abnormalities, proteinuria, and anemia. It is a key role to control these risk factors. However, there is currently a lack of evidence to support that. Future CKD should focus not only on the inherent cells but also on the electrolyte abnormalities, proteinuria, and anemia. Although creatinine levels, proteinuria, urea nitrogen, and physiological and biochemical parameters could reflect renal functions in part, these outcomes might not show a replaceable role in the progression of renal diseases (Luo et al., 2019; Kurth et al., 2020). Therefore, evaluating the composite of renal outcomes in clinical trials is a future direction for researchers. Performing well-designed, randomized, placebo-controlled clinical trials for ginsenosides in humans are very urgent and pivotal.

Furthermore, oral bioavailability has been reported to affect the efficacy of some drugs. It has been demonstrated that the majority of ginsenosides had low oral bioavailability, which limits their long-term efficacy and stability (Kim et al., 2014; Biswas et al., 2017; Chen et al., 2018; Jin et al., 2018). Some scholars tend to explain the factors that lead to low oral bioavailability of ginsenosides, which large molecular weight (Han et al., 2006), low water-solubility (Liu et al., 2009), poor gastrointestinal stability of ginsenosides (Artursson et al., 1993), and intestinal or hepatic first-pass effect, then leading to low oral bioavailability (Yanni, 2007). Future studies should address these issues to fully determine the bioavailability of ginsenosides *via* building effective drug delivery systems, including vesicles (Chen et al., 2014), microsphere (Wei et al., 2007), micelles (Xiong et al., 2008), emulsion delivery systems (Li et al., 2015b), and nanoparticle drug delivery systems (Cai et al., 2014).

The safety of Panax ginseng C.A.Mey. in clinical practices is an issue of widespread concern. Although the efficacy and safety of Panax ginseng C.A.Mey. or ginsenosides have been confirmed in these clinical trials in Table X, the safety should be evaluated with caution due to the low quality and quantity of research. Moreover, natural products are the basis of traditional Chinese medicine, which the most biological function is entirely by identifying their pharmacologically active ingredients. Hence, it is difficult to ensure safety (Li et al., 2020a). In clinical applications, possible side effects need much-weighted attention.

Evidence that ginsenosides have significant effects on anti-oxidative, anti-inflammatory, anti-apoptotic, and anti-fibrosis, with different molecular mechanisms. These processes participate in the regulation of physiological and pathological conditions and are implicated in kidney disease development (Kim et al., 2019; Knoppert et al., 2019; Alicic et al., 2021). However, a comprehensive understanding of the mechanisms that ginsenosides regulate in diverse pathological conditions is lacking. Additionally, more in-depth research should explore the detailed molecular mechanisms of action and pharmacokinetic profile *in vivo*. Careful study will be required to resolve these issues in the future.

## Conclusion

**FIGURE 3:**
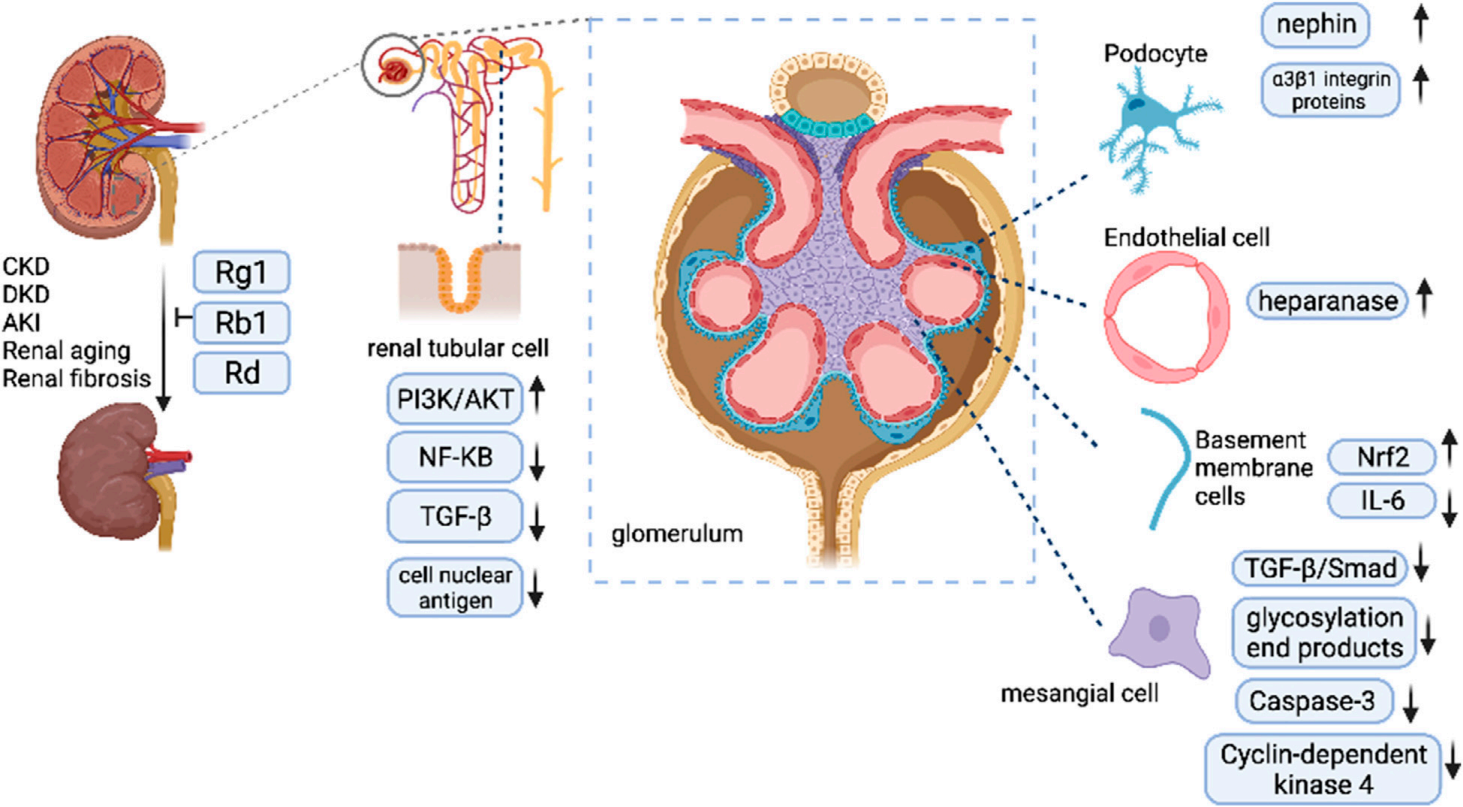
Ginsenosides exert their kidney-protected effects.

In this work, we summarize brief information on diverse types of studies *in vitro* and vivo concerning kidney diseases that suggest similar pathological mechanisms, including inflammatory response, apoptosis of innate renal cells, vascular endothelial function, renal senescence, and renal fibrosis. These pathological processes have been identified, all of which might be influenced by ginsenosides. In clinical applications, its efficacy has been demonstrated in several clinical trials. As an advantage to anti-inflammatory, ginsenosides can improve glomerular endothelial barrier function; alleviate the apoptosis of renal cells induced by ischemia and reperfusion to protect the kidney function in AKI; inhibit the excessive accumulation of ECM, including collagen and fibronectin as well as fibrotic markers, especially TGF-β1 in renal tubular cells; inhibit apoptosis of glomerular mesangial cells; decrease the damage to podocyte cells, including apoptotic and necrotic changes. Ginsenosides exert their kidney-protected effects in the kidney (Figure 3). Collectively, ginsenosides are capable of being a remedy for kidney protection.

Effective therapeutics is currently available for this situation in several clinical trials. However, most of these studies reported contradictory or conflicting findings because of low methodological quality. For this reason, strong evidence is still needed to solve this issue, such as RCTs of rigorous trial design methods and a large, multicenter sample. Moreover, abundant studies have repetitively revealed the therapeutic potential of ginsenosides in kidney-associated human diseases. Meanwhile, new guidelines about Panax ginseng C.A.Mey. usage are imperative to guarantee safety and effectiveness, which is vital for standardized operation practice and experimentally controlled methods. This review provides the first systematic summary of studies examining the role of kidney protection, which will be helpful in the development of kidney-related diseases therapy in the future and gain more reliable and reproducible data.

## References

[B1] Alaofi A. L. (2020). Sinapic Acid Ameliorates the Progression of Streptozotocin (STZ)-Induced Diabetic Nephropathy in Rats via NRF2/HO-1 Mediated Pathways. Front. Pharmacol. 11, 1119. 10.3389/fphar.2020.01119 32792955 PMC7390867

[B2] Alicic R. Z. Cox E. J. Neumiller J. J. Tuttle K. R. (2021). Incretin Drugs in Diabetic Kidney Disease: Biological Mechanisms and Clinical Evidence. Nat. Rev. Nephrol. 17, 227–244. 10.1038/s41581-020-00367-2 33219281

[B3] Artursson P. Ungell A. L. Löfroth J. E. (1993). Selective Paracellular Permeability in Two Models of Intestinal Absorption: Cultured Monolayers of Human Intestinal Epithelial Cells and Rat Intestinal Segments. Pharm. Res. 10, 1123–1129. 10.1023/a:1018903931777 8415396

[B4] Bai L. Gao J. Wei F. Zhao J. Wang D. Wei J. (2018). Therapeutic Potential of Ginsenosides as an Adjuvant Treatment for Diabetes. Front. Pharmacol. 9, 423. 10.3389/fphar.2018.00423 29765322 PMC5938666

[B5] Barrera-Chimal J. Jaisser F. (2020). Pathophysiologic Mechanisms in Diabetic Kidney Disease: A Focus on Current and Future Therapeutic Targets. Diabetes Obes. Metab. 22 Suppl 1, 16–31. 10.1111/dom.13969 32267077

[B6] Biswas T. Mathur A. K. Mathur A. (2017). A Literature Update Elucidating Production of Panax Ginsenosides with a Special Focus on Strategies Enriching the Anti-neoplastic Minor Ginsenosides in Ginseng Preparations. Appl. Microbiol. Biotechnol. 101, 4009–4032. 10.1007/s00253-017-8279-4 28411325

[B7] Blaine J. Dylewski J. (2020). Regulation of the Actin Cytoskeleton in Podocytes. Cells 9, 1700. 10.3390/cells9071700 PMC7408282 32708597

[B8] Cai H. Wen X. Wen L. Tirelli N. Zhang X. Zhang Y. (2014). Enhanced Local Bioavailability of Single or Compound Drugs Delivery to the Inner Ear through Application of PLGA Nanoparticles via Round Window Administration. Int. J. Nanomed. 9, 5591–5601. 10.2147/IJN.S72555 PMC4257110 25489245

[B9] Cao M. Yan H. Han X. Weng L. Wei Q. Sun X. (2019). Ginseng-derived Nanoparticles Alter Macrophage Polarization to Inhibit Melanoma Growth. J. Immunother. Cancer 7, 326. 10.1186/s40425-019-0817-4 31775862 PMC6882204

[B10] Chang-Xiao L. Pei-Gen X. (1992). Recent Advances on Ginseng Research in China. J. Ethnopharmacol. 36, 27–38. 10.1016/0378-8741(92)90057-X 1501490

[B11] Chen D. Yu H. Mu H. Li G. Shen Y. (2014). Novel Multicore Niosomes Based on Double pH-Sensitive Mixed Micelles for Ginsenoside Rh2 Delivery. Artif. Cell Nanomed. Biotechnol. 42, 205–209. 10.3109/21691401.2013.794358 24823243

[B12] Chen J. Li M. Chen L. Wang Y. Li S. Zhang Y. (2018). Effects of Processing Method on the Pharmacokinetics and Tissue Distribution of Orally Administered Ginseng. J. Ginseng Res. 42, 27–34. 10.1016/j.jgr.2016.12.008 29348719 PMC5766692

[B13] Chen T. K. Knicely D. H. Grams M. E. (2019). Chronic Kidney Disease Diagnosis and Management: A Review. JAMA 322, 1294–1304. 10.1001/jama.2019.14745 31573641 PMC7015670

[B14] Chen S. J. Lv L. L. Liu B. C. Tang R. N. (2020). Crosstalk between Tubular Epithelial Cells and Glomerular Endothelial Cells in Diabetic Kidney Disease. Cell Prolif. 53, e12763. 10.1111/cpr.12763 31925859 PMC7106959

[B15] Docherty M. H. O'Sullivan E. D. Bonventre J. V. Ferenbach D. A. (2019). Cellular Senescence in the Kidney. J. Am. Soc. Nephrol. 30, 726–736. 10.1681/ASN.2018121251 31000567 PMC6493983

[B16] Doh K. C. Lim S. W. Piao S. G. Jin L. Heo S. B. Zheng Y. F. (2013). Ginseng Treatment Attenuates Chronic Cyclosporine Nephropathy via Reducing Oxidative Stress in an Experimental Mouse Model. Am. J. Nephrol. 37, 421–433. 10.1159/000349921 23594788

[B17] Doshi S. M. Friedman A. N. (2017). Diagnosis and Management of Type 2 Diabetic Kidney Disease. Clin. J. Am. Soc. Nephrol. 12, 1366–1373. 10.2215/CJN.11111016 28280116 PMC5544517

[B18] Du Y. G. Wang L. P. Qian J. W. Zhang K. N. Chai K. F. (2016). Panax Notoginseng Saponins Protect Kidney from Diabetes by Up-Regulating Silent Information Regulator 1 and Activating Antioxidant Proteins in Rats. Chin. J. Integr. Med. 22, 910–917. 10.1007/s11655-015-2446-1 26712211

[B19] Fan S. Zhang Z. Su H. Xu P. Qi H. Zhao D. (2020a). Panax Ginseng Clinical Trials: Current Status and Future Perspectives. Biomed. Pharmacother. 132, 110832. 10.1016/j.biopha.2020.110832 33059260

[B20] Fan W. Huang Y. Zheng H. Li S. Li Z. Yuan L. (2020b). Ginsenosides for the Treatment of Metabolic Syndrome and Cardiovascular Diseases: Pharmacology and Mechanisms. Biomed. Pharmacother. 132, 110915. 10.1016/j.biopha.2020.110915 33254433

[B21] Fan K. Zeng L. Guo J. Xie S. Yu Y. Chen J. (2021). Visualized Podocyte-Targeting and Focused Ultrasound Responsive Glucocorticoid Nano-Delivery System against Immune-Associated Nephropathy without Glucocorticoid Side Effect. Theranostics 11, 2670–2690. 10.7150/thno.53083 33456566 PMC7806481

[B22] Fang Y. Gong A. Y. Haller S. T. Dworkin L. D. Liu Z. Gong R. (2020). The Ageing Kidney: Molecular Mechanisms and Clinical Implications. Ageing Res. Rev. 63, 101151. 10.1016/j.arr.2020.101151 32835891 PMC7595250

[B23] Fu J. Lee K. Chuang P. Y. Liu Z. He J. C. (2015). Glomerular Endothelial Cell Injury and Cross Talk in Diabetic Kidney Disease. Am. J. Physiol. Ren. Physiol. 308, F287–F297. 10.1152/ajprenal.00533.2014 PMC4329492 25411387

[B24] Gui Q. F. Xu Z. R. Xu K. Y. Yang Y. M. (2016). The Efficacy of Ginseng-Related Therapies in Type 2 Diabetes Mellitus: An Updated Systematic Review and Meta-Analysis. Medicine (Baltimore) 95, e2584. 10.1097/MD.0000000000002584 26871778 PMC4753873

[B25] Guo C. Dong G. Liang X. Dong Z. (2019a). Epigenetic Regulation in AKI and Kidney Repair: Mechanisms and Therapeutic Implications. Nat. Rev. Nephrol. 15, 220–239. 10.1038/s41581-018-0103-6 30651611 PMC7866490

[B26] Guo X. Zhang J. Liu M. Zhao G. C. (2019b). Protective Effect of Ginsenoside Rg1 on Attenuating Anti-GBM Glomerular Nephritis by Activating NRF2 Signalling. Artif. Cell Nanomed. Biotechnol. 47, 2972–2979. 10.1080/21691401.2019.1640712 31322005

[B27] Han M. Sha X. Wu Y. Fang X. (2006). Oral Absorption of Ginsenoside Rb1 Using *In Vitro* and *In Vivo* Models. Planta Med. 72, 398–404. 10.1055/s-2005-916211 16557452

[B28] Hong S. H. Hwang H. J. Kim J. W. Kim J. A. Lee Y. B. Roh E. (2020). Ginsenoside Compound-Mc1 Attenuates Oxidative Stress and Apoptosis in Cardiomyocytes through an AMP-Activated Protein Kinase-dependent Mechanism. J. Ginseng Res. 44, 664–671. 10.1016/j.jgr.2019.08.006 32617047 PMC7322759

[B29] Huang Q. Zhang H. Bai L. P. Law B. Y. K. Xiong H. Zhou X. (2020). Novel Ginsenoside Derivative 20(S)-Rh2E2 Suppresses Tumor Growth and Metastasis *In Vivo* and *In Vitro* via Intervention of Cancer Cell Energy Metabolism. Cell Death Dis. 11, 621. 10.1038/s41419-020-02881-4 32796841 PMC7427995

[B30] Humphreys B. D. (2018). Mechanisms of Renal Fibrosis. Annu. Rev. Physiol. 80, 309–326. 10.1146/annurev-physiol-022516-034227 29068765

[B31] Jang M. Choi J. H. Chang Y. Lee S. J. Nah S. Y. Cho I. H. (2019). Gintonin, a Ginseng-Derived Ingredient, as a Novel Therapeutic Strategy for Huntington's Disease: Activation of the Nrf2 Pathway through Lysophosphatidic Acid Receptors. Brain Behav. Immun. 80, 146–162. 10.1016/j.bbi.2019.03.001 30853569

[B32] Jin Z. H. Qiu W. Liu H. Jiang X. H. Wang L. (2018). Enhancement of Oral Bioavailability and Immune Response of Ginsenoside Rh2 by Co-administration with Piperine. Chin. J. Nat. Med. 16, 143–149. 10.1016/S1875-5364(18)30041-4 29455730

[B33] Jung K. Y. Kim D. S. Oh S. R. Lee I. S. Lee J. J. Park J. D. (1998). Platelet Activating Factor Antagonist Activity of Ginsenosides. Biol. Pharm. Bull. 21, 79–80. 10.1248/bpb.21.79 9477174

[B34] Kalluri R. (2003). Basement Membranes: Structure, Assembly and Role in Tumour Angiogenesis. Nat. Rev. Cancer 3, 422–433. 10.1038/nrc1094 12778132

[B35] Kim W. Y. Kim J. M. Han S. B. Lee S. K. Kim N. D. Park M. K. (2000). Steaming of Ginseng at High Temperature Enhances Biological Activity. J. Nat. Prod. 63, 1702–1704. 10.1021/np990152b 11141123

[B36] Kim E. J. Oh H. A. Choi H. J. Park J. H. Kim D. H. Kim N. J. (2013). Heat-processed Ginseng Saponin Ameliorates the Adenine-Induced Renal Failure in Rats. J. Ginseng Res. 37, 87–93. 10.5142/jgr.2013.37.87 23717161 PMC3659619

[B37] Kim E. O. Cha K. H. Lee E. H. Kim S. M. Choi S. W. Pan C. H. (2014). Bioavailability of Ginsenosides from white and Red Ginsengs in the Simulated Digestion Model. J. Agric. Food Chem. 62, 10055–10063. 10.1021/jf500477n 25175701

[B38] Kim Y. G. Kim S.-M. Kim K.-P. Lee S.-H. Moon J.-Y. (2019). The Role of Inflammasome-dependent and Inflammasome-independent NLRP3 in the Kidney. Cells 8, 1389. 10.3390/cells8111389 PMC6912448 31694192

[B39] Kim J. H. (2012). Cardiovascular Diseases and Panax Ginseng: A Review on Molecular Mechanisms and Medical Applications. J. Ginseng Res. 36, 16–26. 10.5142/jgr.2012.36.1.16 23717100 PMC3659571

[B40] Knoppert S. N. Valentijn F. A. Nguyen T. Q. Goldschmeding R. Falke L. L. (2019). Cellular Senescence and the Kidney: Potential Therapeutic Targets and Tools. Front. Pharmacol. 10, 770. 10.3389/fphar.2019.00770 31354486 PMC6639430

[B41] Koizumi S. Ohsawa K. Inoue K. Kohsaka S. (2013). Purinergic Receptors in Microglia: Functional Modal Shifts of Microglia Mediated by P2 and P1 Receptors. Glia 61, 47–54. 10.1002/glia.22358 22674620

[B42] Kulaputana O. Thanakomsirichot S. Anomasiri W. (2007). Ginseng Supplementation Does Not Change Lactate Threshold and Physical Performances in Physically Active Thai Men. J. Med. Assoc. Thai 90, 1172–1179. 17624213

[B43] Kurth M. J. McBride W. T. McLean G. Watt J. Domanska A. Lamont J. V. (2020). Acute Kidney Injury Risk in Orthopaedic Trauma Patients Pre and post Surgery Using a Biomarker Algorithm and Clinical Risk Score. Sci. Rep. 10, 20005. 10.1038/s41598-020-76929-y 33203963 PMC7673130

[B44] Lang J. Cao H. Wei A. (1998). Comparative Study on Effect of Panax Notoginseng and Ticlid in Treating Early Diabetic Nephropathy. Zhongguo Zhong Xi Yi Jie He Za Zhi 18, 727–729. 11475719

[B45] Leeuwis J. W. Nguyen T. Q. Dendooven A. Kok R. J. Goldschmeding R. (2010). Targeting Podocyte-Associated Diseases. Adv. Drug Deliv. Rev. 62, 1325–1336. 10.1016/j.addr.2010.08.012 20828590

[B46] Li S. S. Ye J. M. Deng Z. Y. Yu L. X. Gu X. X. Liu Q. F. (2015a). Ginsenoside-Rg1 Inhibits Endoplasmic Reticulum Stress-Induced Apoptosis after Unilateral Ureteral Obstruction in Rats. Ren. Fail. 37, 890–895. 10.3109/0886022X.2015.1015427 25707520

[B47] Li T. Shu Y. J. Cheng J. Y. Liang R. C. Dian S. N. Lv X. X. (2015b). Pharmacokinetics and Efficiency of Brain Targeting of Ginsenosides Rg1 and Rb1 Given as Nao-Qing Microemulsion. Drug Dev. Ind. Pharm. 41, 224–231. 10.3109/03639045.2013.858734 24237326

[B48] Li S. S. He A. L. Deng Z. Y. Liu Q. F. (2018). Ginsenoside-Rg1 Protects against Renal Fibrosis by Regulating the Klotho/TGF-β1/Smad Signaling Pathway in Rats with Obstructive Nephropathy. Biol. Pharm. Bull. 41, 585–591. 10.1248/bpb.b17-00934 29607931

[B49] Li X. Mo N. Li Z. (2020a). Ginsenosides: Potential Therapeutic Source for Fibrosis-Associated Human Diseases. J. Ginseng Res. 44, 386–398. 10.1016/j.jgr.2019.12.003 32372860 PMC7195584

[B50] Li X. Zou Y. Xing J. Fu Y.-Y. Wang K.-Y. Wan P.-Z. (2020b). Pretreatment with Roxadustat (FG-4592) Attenuates Folic Acid-Induced Kidney Injury through Antiferroptosis via Akt/GSK-3β/Nrf2 Pathway. Oxid. Med. Cell Longev. 2020, 1–17. 10.1155/2020/6286984 PMC6995323 32051732

[B51] Li Z. Feng H. Han L. Ding L. Shen B. Tian Y. (2020c). Chicoric Acid Ameliorate Inflammation and Oxidative Stress in Lipopolysaccharide and D-Galactosamine Induced Acute Liver Injury. J. Cel. Mol. Med. 24, 3022–3033. 10.1111/jcmm.14935 PMC7077529 31989756

[B52] Li Y. Hou J. G. Liu Z. Gong X. J. Hu J. N. Wang Y. P. (2021). Alleviative Effects of 20(R)-Rg3 on HFD/STZ-induced Diabetic Nephropathy via MAPK/NF-κB Signaling Pathways in C57BL/6 Mice. J. Ethnopharmacol. 267, 113500. 10.1016/j.jep.2020.113500 33091499

[B53] Lim S. W. Doh K. C. Jin L. Jin J. Piao S. G. Heo S. B. (2014). Ginseng Treatment Attenuates Autophagic Cell Death in Chronic Cyclosporine Nephropathy. Nephrology (Carlton) 19, 490–499. 10.1111/nep.12273 24796922

[B54] Lin C. X. Li Y. Liang S. Tao J. Zhang L. S. Su Y. F. (2019). Metformin Attenuates Cyclosporine A-Induced Renal Fibrosis in Rats. Transplantation 103, e285–e296. 10.1097/TP.0000000000002864 31335763

[B55] Liu H. Yang J. Du F. Gao X. Ma X. Huang Y. (2009). Absorption and Disposition of Ginsenosides after Oral Administration of Panax Notoginseng Extract to Rats. Drug Metab. Dispos. 37, 2290–2298. 10.1124/dmd.109.029819 19786509

[B56] Liu Q. F. Deng Z. Y. Ye J. M. He A. L. Li S. S. (2015). Ginsenoside Rg1 Protects Chronic Cyclosporin a Nephropathy from Tubular Cell Apoptosis by Inhibiting Endoplasmic Reticulum Stress in Rats. Transpl. Proc. 47, 566–569. 10.1016/j.transproceed.2014.10.047 25769608

[B57] Liu L. Vollmer M. K. Ahmad A. S. Fernandez V. M. Kim H. Doré S. (2019). Pretreatment with Korean Red Ginseng or Dimethyl Fumarate Attenuates Reactive Gliosis and Confers Sustained Neuroprotection against Cerebral Hypoxic-Ischemic Damage by an Nrf2-dependent Mechanism. Free Radic. Biol. Med. 131, 98–114. 10.1016/j.freeradbiomed.2018.11.017 30458277 PMC6362849

[B58] Liu X. Mi X. Wang Z. Zhang M. Hou J. Jiang S. (2020). Ginsenoside Rg3 Promotes Regression from Hepatic Fibrosis through Reducing Inflammation-Mediated Autophagy Signaling Pathway. Cel. Death Dis. 11, 454. 10.1038/s41419-020-2597-7 PMC7293224 32532964

[B59] Luo S. Coresh J. Tin A. Rebholz C. M. Appel L. J. Chen J. (2019). Serum Metabolomic Alterations Associated with Proteinuria in CKD. Clin. J. Am. Soc. Nephrol. 14, 342–353. 10.2215/CJN.10010818 30733224 PMC6419293

[B60] Matoba K. Takeda Y. Nagai Y. Kawanami D. Utsunomiya K. Nishimura R. (2019). Unraveling the Role of Inflammation in the Pathogenesis of Diabetic Kidney Disease. Int. J. Mol. Sci. 20, 3393. 10.3390/ijms20143393 PMC6678414 31295940

[B61] Nabavi S. F. Sureda A. Habtemariam S. Nabavi S. M. (2015). Ginsenoside Rd and Ischemic Stroke; a Short Review of Literatures. J. Ginseng Res. 39, 299–303. 10.1016/j.jgr.2015.02.002 26869821 PMC4593783

[B62] Nah S.-Y. (1997). Ginseng; Recent Advances and Trends. J. Ginseng Res. 21, 1–12.

[B63] Ni X. J. Xu Z. Q. Jin H. Zheng S. L. Cai Y. Wang J. J. (2017). Ginsenoside Rg1 Protects Human Renal Tubular Epithelial Cells from Lipopolysaccharide-Induced Apoptosis and Inflammation Damage. Braz. J. Med. Biol. Res. 51, e6611. 10.1590/1414-431X20176611 29267498 PMC5731327

[B64] Park M. J. Bae C. S. Lim S. K. Kim D. I. Lim J. C. Kim J. C. (2010). Effect of Protopanaxadiol Derivatives in High Glucose-Induced Fibronectin Expression in Primary Cultured Rat Mesangial Cells: Role of Mitogen-Activated Protein Kinases and Akt. Arch. Pharm. Res. 33, 151–157. 10.1007/s12272-010-2237-3 20191356

[B65] Peng S. L. Guo Z. A. (2010). Effect of Total Saponins of Panax Notoginseng on Urinary Albumin in Patients with Chronic Renal Failure. Zhongguo Wei Zhong Bing Ji Jiu Yi Xue 22, 744–746. 10.3760/cma.j.issn.1003-0603.2010.12.013 21190603

[B66] Podkowińska A. Formanowicz D. (2020). Chronic Kidney Disease as Oxidative Stress- and Inflammatory-Mediated Cardiovascular Disease. Antioxidants (Basel) 9, 752. 10.3390/antiox9080752 PMC7463588 32823917

[B67] Quan L. H. Zhang C. Dong M. Jiang J. Xu H. Yan C. (2020). Myristoleic Acid Produced by Enterococci Reduces Obesity through Brown Adipose Tissue Activation. Gut 69, 1239–1247. 10.1136/gutjnl-2019-319114 31744910

[B68] Rastogi V. Santiago-Moreno J. Doré S. (2014). Ginseng: a Promising Neuroprotective Strategy in Stroke. Front Cel. Neurosci. 8, 457. 10.3389/fncel.2014.00457 PMC4299449 25653588

[B69] Shao J.-W. Jiang J.-L. Zou J.-J. Yang M.-Y. Chen F.-M. Zhang Y.-J. (2020). Therapeutic Potential of Ginsenosides on Diabetes: From Hypoglycemic Mechanism to Clinical Trials. J. Funct. Foods 64, 103630. 10.1016/j.jff.2019.103630

[B70] Shen C. Y. Jiang J. G. Yang L. Wang D. W. Zhu W. (2017). Anti-ageing Active Ingredients from Herbs and Nutraceuticals Used in Traditional Chinese Medicine: Pharmacological Mechanisms and Implications for Drug Discovery. Br. J. Pharmacol. 174, 1395–1425. 10.1111/bph.13631 27659301 PMC5429334

[B71] Shi Y. Gao Y. Wang T. Wang X. He J. Xu J. (2020). Ginsenoside Rg1 Alleviates Podocyte EMT Passage by Regulating AKT/GSK3 β/β-Catenin Pathway by Restoring Autophagic Activity. Evid. Based Complement. Alternat Med. 2020, 1903627. 10.1155/2020/1903627 32082395 PMC7011395

[B72] Singbartl K. Kellum J. A. (2012). AKI in the ICU: Definition, Epidemiology, Risk Stratification, and Outcomes. Kidney Int. 81, 819–825. 10.1038/ki.2011.339 21975865

[B73] Srour B. Fezeu L. K. Kesse-Guyot E. Allès B. Debras C. Druesne-Pecollo N. (2020). Ultraprocessed Food Consumption and Risk of Type 2 Diabetes Among Participants of the NutriNet-Santé Prospective Cohort. JAMA Intern. Med. 180, 283–291. 10.1001/jamainternmed.2019.5942 31841598 PMC6990737

[B74] Stavro P. M. Woo M. Leiter L. A. Heim T. F. Sievenpiper J. L. Vuksan V. (2006). Long-term Intake of North American Ginseng Has No Effect on 24-hour Blood Pressure and Renal Function. Hypertension 47, 791–796. 10.1161/01.HYP.0000205150.43169.2c 16520410

[B75] Stenvinkel P. Meyer C. J. Block G. A. Chertow G. M. Shiels P. G. (2020). Understanding the Role of the Cytoprotective Transcription Factor Nuclear Factor Erythroid 2-related Factor 2-lessons from Evolution, the Animal Kingdom and Rare Progeroid Syndromes. Nephrol. Dial. Transpl. 35, 2036–2045. 10.1093/ndt/gfz120 PMC7716811 31302696

[B111] Sun Y.-J. Chen H. Hao Z.-Y. Wang J.-M. Zhang Y.-L. Zhao X. (2014). Chemical constituents from fruit of Panax ginseng. Zhong Yao Cai 37, 1387–1390. 25726647

[B76] Sun W. Feng L. Y. Zhao Z. J. Liu T. H. Yang M. J. (2011). Study on Antioxidant Effects and Inhibition of Podocyte Apoptosis of PNS on DN Rat. China J. Tradit. Chin. Med. Pharm. 26 (5), 1062–1067. Available at: http://med.wanfangdata.com.cn/Paper/Detail?id=PeriodicalPaper_zgyyxb201105039 (Accessed May 21, 2021).

[B77] Sun Q. Meng Q. T. Jiang Y. Liu H. M. Lei S. Q. Su W. T. (2013). Protective Effect of Ginsenoside Rb1 against Intestinal Ischemia-Reperfusion Induced Acute Renal Injury in Mice. PLoS One 8, e80859. 10.1371/journal.pone.0080859 24324637 PMC3851764

[B78] Sun Q. Xia Y. Z. Jiang Y. (2018). Ginsenoside Rb1 Attenuates Intestinal Ischemia-Reperfusion Induced Renal Injury. Med. J. Wuhan Univ. 39 (3), 385–388,393. 10.14188/j.1671-8852.2018.6008 Available at: https://www.cnki.com.cn/Article/CJFDTotal-HBYK201803009.htm (Accessed June 3, 2021).

[B79] Tang X. Y. Liu G. L. Xia Z. K. Xu H. Q. (2013). Effect of Ginsenoside Rg1 on Driamycin-Induced Apoptosis of Mesangial Cell. Mod. Med. (4), 226–231. 10.3969/j.issn.1671-7562.2013.04.002

[B80] Thomas M. C. Brownlee M. Susztak K. Sharma K. Jandeleit-Dahm K. A. Zoungas S. (2015). Diabetic Kidney Disease. Nat. Rev. Dis. Primers 1, 15018. 10.1038/nrdp.2015.18 27188921 PMC7724636

[B81] Tonelli M. Dickinson J. A. (2020). Early Detection of CKD: Implications for Low-Income, Middle-Income, and High-Income Countries. J. Am. Soc. Nephrol. 31, 1931–1940. 10.1681/ASN.2020030277 32839279 PMC7461685

[B82] Wan S. Wan S. Jiao X. Cao H. Gu Y. Yan L. (2021). Advances in Understanding the Innate Immune-Associated Diabetic Kidney Disease. FASEB J. 35, e21367. 10.1096/fj.202002334R 33508160 PMC12315487

[B83] Wang G. Q. (2011). Evaluation of the Antidiabetic Effect of Ginsenoside RB1 Based on FFA Spillover of Adipocytes. Jiangsu: Nanjing University of Traditional Chinese Medicine. 10.7666/d.y1945370

[B84] Webster A. C. Nagler E. V. Morton R. L. Masson P. (2017). Chronic Kidney Disease. Lancet 389, 1238–1252. 10.1016/S0140-6736(16)32064-5 27887750

[B85] Wei H. J. Yang H. H. Chen C. H. Lin W. W. Chen S. C. Lai P. H. (2007). Gelatin Microspheres Encapsulated with a Nonpeptide Angiogenic Agent, Ginsenoside Rg1, for Intramyocardial Injection in a Rat Model with Infarcted Myocardium. J. Control. Release 120, 27–34. 10.1016/j.jconrel.2007.04.005 17532519

[B86] Wong A. S. Che C. M. Leung K. W. (2015). Recent Advances in Ginseng as Cancer Therapeutics: a Functional and Mechanistic Overview. Nat. Prod. Rep. 32, 256–272. 10.1039/C4NP00080C 25347695

[B87] Wu S. P. Wang Y. D. (2015). Protection of Ginsenoside Rg3 on Oxidative Stress Injury of Human Glomerular Mesangial Cells Induced by H2O2 and its Mechanism. Drugs Clinic 30, 1437–1442. 10.7501/j.issn.1674-5515.2015.12.002

[B88] Wu Q. Wang X. Nepovimova E. Wang Y. Yang H. Kuca K. (2018). Mechanism of Cyclosporine A Nephrotoxicity: Oxidative Stress, Autophagy, and Signalings. Food Chem. Toxicol. 118, 889–907. 10.1016/j.fct.2018.06.054 29960018

[B89] Xie X. S. Yang M. Liu H. C. Zuo C. Li Z. Deng Y. (2008). Influence of Ginsenoside Rg1, a Panaxatriol Saponin from Panax Notoginseng, on Renal Fibrosis in Rats with Unilateral Ureteral Obstruction. J. Zhejiang Univ. Sci. B 9, 885–894. 10.1631/jzus.B0820024 18988308 PMC2579952

[B90] Xie X. S. Liu H. C. Yang M. Zuo C. Deng Y. Fan J. M. (2009a). Ginsenoside Rb1, a Panoxadiol Saponin against Oxidative Damage and Renal Interstitial Fibrosis in Rats with Unilateral Ureteral Obstruction. Chin. J. Integr. Med. 15, 133–140. 10.1007/s11655-009-0133-9 19407952

[B91] Xie X. S. Yang M. Liu H. C. Zuo C. Li H. J. Fan J. M. (2009b). Ginsenoside Rg1, a Major Active Component Isolated from Panax Notoginseng, Restrains Tubular Epithelial to Myofibroblast Transition *In Vitro* . J. Ethnopharmacol. 122, 35–41. 10.1016/j.jep.2008.11.020 19101622

[B92] Xie L. Zhai R. Chen T. Gao C. Xue R. Wang N. (2020). Panax Notoginseng Ameliorates Podocyte EMT by Targeting the Wnt/β-Catenin Signaling Pathway in STZ-Induced Diabetic Rats. Drug Des. Devel Ther. 14, 527–538. 10.2147/DDDT.S235491 PMC7008200 32103895

[B93] Xiong J. Guo J. Huang L. Meng B. Ping Q. (2008). Self-micelle Formation and the Incorporation of Lipid in the Formulation Affect the Intestinal Absorption of Panax Notoginseng. Int. J. Pharm. 360, 191–196. 10.1016/j.ijpharm.2008.04.016 18502060

[B94] Xu X. Lu Q. Wu J. Li Y. Sun J. (2017). Impact of Extended Ginsenoside Rb1 on Early Chronic Kidney Disease: a Randomized, Placebo-Controlled Study. Inflammopharmacology 25, 33–40. 10.1007/s10787-016-0296-x 27853891

[B95] Xu Y. Wang N. Tan H. Y. Li S. Zhang C. Zhang Z. (2020). Panax Notoginseng Saponins Modulate the Gut Microbiota to Promote Thermogenesis and Beige Adipocyte Reconstruction via Leptin-Mediated AMPKα/STAT3 Signaling in Diet-Induced Obesity. Theranostics 10, 11302–11323. 10.7150/thno.47746 33042284 PMC7532683

[B96] Xue H. Zhao Z. Lin Z. Geng J. Guan Y. Song C. (2019). Selective Effects of Ginseng Pectins on Galectin-3-Mediated T Cell Activation and Apoptosis. Carbohydr. Polym. 219, 121–129. 10.1016/j.carbpol.2019.05.023 31151509

[B97] Yang H. L. Thiyagarajan V. Shen P. C. Mathew D. C. Lin K. Y. Liao J. W. (2019). Anti-EMT Properties of CoQ0 Attributed to PI3K/AKT/NFKB/MMP-9 Signaling Pathway through ROS-Mediated Apoptosis. J. Exp. Clin. Cancer Res. 38, 186. 10.1186/s13046-019-1196-x 31068208 PMC6505074

[B98] Yang C. Wang H. Zhao X. Matsushita K. Coresh J. Zhang L. (2020). CKD in China: Evolving Spectrum and Public Health Implications. Am. J. Kidney Dis. 76, 258–264. 10.1053/j.ajkd.2019.05.032 31492486

[B99] Yanni S. Thakker D. R. 2007). Prodrugs: Absorption, Distribution, Metabolism, Excretion (ADME) Issues, 1043, 1081. 10.1007/978-0-387-49785-3_29

[B100] Yokozawa T. Liu Z. W. Dong E. (1998). A Study of Ginsenoside-Rd in a Renal Ischemia-Reperfusion Model. Nephron. 78, 201–206. 10.1159/000044911 9496738

[B101] Yoon J. J. Lee H. K. Kim H. Y. Han B. H. Lee H. S. Lee Y. J. (2020). Sauchinone Protects Renal Mesangial Cell Dysfunction against Angiotensin II by Improving Renal Fibrosis and Inflammation. Int. J. Mol. Sci. 21, 7003. 10.3390/ijms21197003 PMC7583825 32977573

[B102] Zhang X. K. Zhao Z. J. Cui X. M. Zhang X. X. Yang M. J. (2008). Effect of Ginsenoside-Rg1 and Rb1 on the Kidney and Renal Expression of MCP-1 mRNA and Protein in Rat Model with Diabetic Nephropathy. Chin. J. Integr. Tradit. West. Med. 9 (7), 578–581. 10.3969/j.issn.1009-587X.2008.07.005

[B103] Zhang Y. Yu L. Cai W. Fan S. Feng L. Ji G. (2014). Protopanaxatriol, a Novel PPARγ Antagonist from Panax Ginseng, Alleviates Steatosis in Mice. Sci. Rep. 4, 7375. 10.1038/srep07375 25487878 PMC4260220

[B104] Zhao L. Lan L. G. Min X. L. Lu A. H. Zhu L. Q. He X. H. (2007). Integrated Treatment of Traditional Chinese Medicine and Western Medicine for Early- and Intermediate-Stage Diabetic Nephropathy. Nan Fang Yi Ke Da Xue Xue Bao 27, 1052–1055. 17666348

[B105] Zhao Z. J. Zhang X. K. Zhang X. X. Cui M. X. Yang M. J. (2008). Protective Effect of Rg L and Rb L on Kidney in Rats with Diabetic Nephropathy and Their Influences on Expressions of TGF-131mRNA and Protein of Renal Tissue. J. Beijing Univ. Tradit. Chin. Med. 31 (6), 373–377. 10.3321/j.issn:1006-2157.2008.06.00310.3321/j.issn:1006-2157.2008.06.003

[B106] Zhao J. (2018). Effects of Ginsenoside Rg1 on Glomerular Mesangial Cell Inflammation Induced by Glycosylation End Products and TGFβ/Smad Signaling Pathway. China Pharmacist 21 (11), 1919–1923. 10.3321/j.issn:1673-4254.2007.07.037 Available at: http://www.cnki.com.cn/Article/CJFDTotal-ZYSG201811007.htm (Accessed May 25, 2021).

[B107] Zhou J. X. Ai Z. M. Sun W. Wu L. L. Qin L. Li J. (2014). Mechanism Study of Panax Notoginseng Saponins on Protective Effect for Podocyte in Diabetic Nephropathy Mice. China J. Tradit. Chin. Med. Pharm. 29 (05), 1316–1321. Available at: http://med.wanfangdata.com.cn/Paper/Detail?id=PeriodicalPaper_zgyyxb201105039. (Accessed May 21, 2021).

[B108] Zhu M. X. Ran B. Feng Z. Q. Pan Q. W. (2009). Effects of Rb1 and Rg1 on the Expression of Bcl-2, Bax in Apoptosis of HK-2 Cells Induced by the Serum of Kidney Ischemia/reperfusion. Zhongguo Ying Yong Sheng Li Xue Za Zhi 25, 496–499. 21158042

[B109] Zhu T. Wang H. Wang L. Zhong X. Huang W. Deng X. (2019). Ginsenoside Rg1 Attenuates High Glucose induced Endothelial Barrier Dysfunction in Human Umbilical Vein Endothelial Cells by Protecting the Endothelial Glycocalyx. Exp. Ther. Med. 17, 3727–3733. 10.3892/etm.2019.7378 30988758 PMC6447798

[B110] Zhu Y. Zhu C. Yang H. Deng J. Fan D. (2020). Protective Effect of Ginsenoside Rg5 against Kidney Injury via Inhibition of NLRP3 Inflammasome Activation and the MAPK Signaling Pathway in High-Fat Diet/streptozotocin-Induced Diabetic Mice. Pharmacol. Res. 155, 104746. 10.1016/j.phrs.2020.104746 32156650

